# Differentiation of *Bacillus cereus* and *Bacillus thuringiensis* Using Genome-Guided MALDI-TOF MS Based on Variations in Ribosomal Proteins

**DOI:** 10.3390/microorganisms10050918

**Published:** 2022-04-27

**Authors:** Minling Chen, Xianhu Wei, Junhui Zhang, Huan Zhou, Nuo Chen, Juan Wang, Ying Feng, Shubo Yu, Jumei Zhang, Shi Wu, Qinghua Ye, Rui Pang, Yu Ding, Qingping Wu

**Affiliations:** 1 School of Bioscience and Bioengineering, South China University of Technology, Guangzhou 510006, China; cmling0101@163.com; 2 Guangdong Provincial Key Laboratory of Microbial Safety and Health, State Key Laboratory of Applied Microbiology Southern China, Institute of Microbiology, Guangdong Academy of Sciences, Guangzhou 510070, China; wxhu7508@163.com (X.W.); wangjuan@scau.edu.cn (J.W.); fengying717@163.com (Y.F.); yushubo5663860@163.com (S.Y.); zhangjm@gdim.cn (J.Z.); wushiloveyou@126.com (S.W.); yeqinghua2002@163.com (Q.Y.); pr839@163.com (R.P.); 3 Department of Food Science and Technology, Jinan University, Guangzhou 510632, China; conanjunhui@163.com (J.Z.); zh71739601@163.com (H.Z.); chennuo7676@163.com (N.C.)

**Keywords:** *Bacillus cereus*, *Bacillus thuringiensis*, genome-guided MALDI-TOF MS, ribosomal protein, high-throughput differentiation

## Abstract

*Bacillus cereus* and *B. thuringiensis* are closely related species that are relevant to foodborne diseases and biopesticides, respectively. Unambiguous differentiation of these two species is crucial for bacterial taxonomy. As genome analysis offers an objective but time-consuming classification of *B. cereus* and *B. thuringiensis*, in the present study, matrix-assisted laser desorption ionization-time of flight mass spectrometry (MALDI-TOF MS) was used to accelerate this process. By combining in silico genome analysis and MALDI-TOF MS measurements, four species-specific peaks of *B. cereus* and *B. thuringiensis* were screened and identified. The species-specific peaks of *B. cereus* were m/z 3211, 6427, 9188, and 9214, and the species-specific peaks of *B. thuringiensis* were m/z 3218, 6441, 9160, and 9229. All the above peaks represent ribosomal proteins, which are conserved and consistent with the phylogenetic relationship between *B. cereus* and *B. thuringiensis*. The specificity of the peaks was robustly verified using common foodborne pathogens. Thus, we concluded that genome-guided MALDI-TOF MS allows high-throughput differentiation of *B. cereus* and *B. thuringiensis* and provides a framework for differentiating other closely related species.

## 1. Introduction

The *Bacillus cereus* group comprises many genetically highly related species, including *B. cereus* and *B. thuringiensis* [1]. These species are unavoidable in food production and processing because of their capability to form resistant spores as well as their ubiquitous distribution in the environment [2]. *B. cereus* is an opportunistic foodborne pathogen that can cause emetic and diarrheal symptoms owing to the production of cereulide and enterotoxins, respectively [3]. It has been estimated that *B. cereus* causes 63,400 foodborne illness cases in the United States every year [4]. From 2006 to 2016, *B. cereus* ranked third in the prevalence of foodborne pathogens in China [5]. *B. thuringiensis* is widely used as a biopesticide because of its ability to produce insecticidal proteins, including crystal (Cry) and cytolytic (Cyt) δ-endotoxins [6,7]. Recent studies have shown that *B. thuringiensis* can also be used to promote plant growth [8], produce metal nanoparticles [9], and biodegrade environmental pollutants [10]. Although *B. cereus* and *B. thuringiensis* have different pathogenicity and applications, they are genetically closely related, and there is still no available ISO to reliably distinguish between them [1]. Accurate taxonomy of *B. cereus* and *B. thuringiensis* is of fundamental importance for phylogenetic taxonomy research, public health, food industry safety, biopesticide market, and biotechnology development.

Great efforts have been made to differentiate *B. cereus* and *B. thuringiensis*, however, the conventional method for differentiation between *B. cereus* and *B. thuringiensis* based on insecticidal crystal proteins has certain limitations, as the genome data show that insecticidal crystal protein genes are not unique to *B. thuringiensis* [11,12,13]. General molecular typing also cannot achieve reliable differentiation, as 16S rRNA gene sequences of *B. cereus* and *B. thuringiensis* share more than 99% similarity [14], and pulsed-field gel electrophoresis also cannot effectively differentiate between *B. cereus* and *B. thuringiensis* [15]. Several molecular targets were screened to identify strains within the *B. cereus* group [16,17,18], and among them, the *xre* gene showed specificity for detecting *B. thuringiensis* [16,18].

In the post-genomic era, whole genome sequencing has been increasingly used for bacterial taxonomy [19]. With the advantage of being reliable, reproducible, and objective [20], genome-based taxonomy offers phylogenetically consistent resolution for classifying bacterial species [21]. The Genome Taxonomy Database (GTDB) was established based on the genome phylogeny of 120 ubiquitous single-copy proteins and provided a standardized taxonomy for bacterial species, including *B. cereus* and *B. thuringiensis* [21]. The genome blast distance phylogeny approach was used to calculate intergenomic distances [22] and clearly separated *B. cereus* and *B. thuringiensis* [23]. In the same study, the phylogenetic tree of the housekeeping gene *pycA* (encoding pyruvate carboxylase) was found to resolve the confusion between *B. cereus* and *B. thuringiensis* [23].

Although genome-based taxonomy assists classification with high resolution, it is still time-consuming, expensive, and labor-intensive, and is not suitable for routine practice. Matrix-assisted laser desorption ionization-time of flight mass spectrometry (MALDI-TOF MS) has been extensively applied in clinical diagnosis [24], food safety control [25,26], and environmental monitoring [27], offering reliable, rapid, and cost-effective microbial identification. In recent studies, MALDI-TOF MS showed sufficient resolution to differentiate between five predominant serovars of the non-typhoidal *Salmonella* [28], O157, O26, and O111 serotypes of *Escherichia coli* [29], different species of the genus *Listeria* [30], and lineages of methicillin-resistant *Staphylococcus aureus* [31]. Meanwhile, MALDI-TOF MS had also been applied to differentiate strains within *B. cereus* group [32,33]. Using MALDI-TOF MS, it is possible to detect ribosomal proteins [34], which are ideal targets for phylogenetic analysis. Proteotyping based on ribosomal proteins provided congruent resolution in comparison to molecular typing methods such as multilocus sequence typing (MLST) [35,36]. By using ribosomal multilocus sequence typing (rMLST) based on 53 ribosomal protein genes, the taxonomic status of diverse species, including *B. cereus* and *B. thuringiensis*, was unambiguously classified [37]. In the present study, as ribosomal proteins are dependable biomarkers in MALDI-TOF MS analysis for species identification, we first screened for robust variations in ribosomal proteins between *B. cereus* and *B. thuringiensis* based on genome analysis and then correlated them with species-specific features in MALDI-TOF MS profiling. The aim of this study was to develop a high-throughput MALDI-TOF MS-based tool for differentiating between *B. cereus* and *B. thuringiensis*.

## 2. Materials and Methods

### 2.1. Bacterial Strains

Two reference strains (*B. cereus* ATCC 14,579 and *B. thuringiensis* ATCC 10792) and 79 strains of *Bacillus* species isolated in our laboratory [38,39,40,41] were selected to screen for potential biomarkers. Detailed information on the strains are provided in Table S1. Thirteen common foodborne pathogens were used to verify the specificity of the biomarkers.

### 2.2. Genomic Data Mining

The genome sequences of 106 *B. cereus* and 175 *B. thuringiensis* strains (Table S2) were downloaded from the National Center for Biotechnology Information (NCBI) database to mine potential biomarkers. The definite classification of selected strains was determined using the GTDB (https://gtdb.ecogenomic.org/, accessed on 1 January 2021) and rMLST databases (https://pubmlst.org/rmlst, accessed on 1 January 2021). The genome sequences were annotated using the Prokka software [42]. The output GFF files were used to perform genome alignment using Roary [43] with an 80% sequence identity cut-off. Aligned sequences of ribosomal proteins were obtained using TBtools [44]. Theoretical molecular weights of ribosomal proteins were calculated from translated amino acid sequences using the web-based compute pI/Mw tool (https://web.expasy.org/compute_pi/, accessed on 15 January 2021) and protein molecular weight tool (http://www.detaibio.com/sms2/protein_mw.html, accessed on 15 January 2021). When the second residue in the amino acid chain was Gly, Ala, Pro, Ser, Thr, Val, or Cys, molecular weights were calculated by considering the N-terminal methionine loss.

### 2.3. Species Identification Based on Phylogenetic Tree of pycA

Genomic DNA was extracted using an ultrasonic-assisted method. Briefly, the bacterial colony suspended in 50 μL of ddH_2_O was treated with an ultrasonic bath operating at 40 kHz for 5 min. The suspension was centrifuged at 15,000× *g* for 2 min. Then, 1 μL of supernatant was used for the following PCR reaction, the reaction mixture contained 12.5 μL of Premix Taq™ (TaKaRa Taq™ Version 2.0 plus dye, Guangzhou, China), 1 μL of each primers (10 μM), 1 μL of DNA template, and 9.5. μL of sterile water. PCR amplification of the *pycA* gene was carried out using the following primers: forward primer, 5′-GTGAAAGCAAGAACACAAGC-3′, and reverse primer, 5′-ATAGTTTTTGTATCCAACTGCG-3′. PCR reactions were conducted as follows: one cycle of initial denaturation at 98 °C for 3 min, followed by 35 cycles of 98 °C for 10 s, 55 °C for 30 s, and 72 °C for 100 s, and a final extension at 72 °C for 10 min. The PCR products were purified and sequenced by Tianyi Huiyuan Bioscience & Technology Inc. (Guangzhou, China).

Identification of *B. cereus* and *B. thuringiensis* was performed as described previously [45]. Briefly, the obtained sequences were aligned with the *pycA* genes of *B. cereus* group reference strains in Liu’s study [23], after which a phylogenetic analysis using the maximum likelihood algorithm was performed with the MEGA X software [46]. Visualization of the phylogenetic tree was performed using iTOL [47].

### 2.4. MALDI-TOF MS Analysis

For MALDI-TOF MS analysis, *Campylobacter jejuni* was inoculated in Skirrow agar plates (Guangdong Huankai Co., Ltd., Guangzhou, China), *Vibrio parahaemolyticus* was inoculated in the Luria-Bertani (LB) agar (Guangdong Huankai Co., Ltd.,) with 3% NaCl, and the other strains were inoculated in the LB agar. All strains were incubated at 37 °C for 16 h. Bacterial colonies were smeared directly onto a 96-well MALDI target plate (Bruker Daltonics, Bremen, Germany) using sterile toothpicks and overlaid with 1 μL of 70% formic acid. After air-drying, 1 μL of the matrix solution containing 10 mg/mL of α-cyano-4-hydroxy-cinnamic acid (HCCA) in 50% (*v*/*v*) acetonitrile with 2.5% (*v*/*v*) trifluoroacetic acid was applied and allowed to dry. The detection was carried out on three different days in quadruplicates for each strain.

The mass spectra were acquired using a Microflex LT/SH smart MALDI-TOF MS (Bruker Daltonics, Bremen, Germany) equipped with a 200 Hz smartbeam solid-state laser and operated in positive linear mode. Mass spectra were automatically recorded within a mass range of 2–20 kDa with a total of 240 laser shots. A bacterial test standard (Bruker Daltonics, Bremen, Germany) was used for external spectral calibration before every experiment.

### 2.5. Data Processing and Identification of Biomarker Proteins

Raw mass spectra were converted to mzML files using the R package MALDIquant [48]. The output mzML files were imported into the Mass-Up software (Mass-Up, Vigo, Spain) for data processing [49]. Mass-Up provided intensity transformation (square root method), spectral smoothing (Savitzky-Golay method), baseline correction (TopHat method), standardization (Total Ion Current method), and peak detection (MassSpecWavelet method; SNR = 6, PeakScaleRange = 2, amp.Th = 0.0001). Output peak lists from the replicates of each strain were used to construct intra- and inter-sample matching using the forward method (tolerance = 300 ppm, reference type = AVG). Species-specific peaks were screened using the biomarker discovery module. Biomarker identification was performed by comparing the species-specific m/z against the theoretical m/z of differential ribosomal proteins in the above genome analysis. The identification result was further validated by searching against proteins below 10,000 Da from genome analysis and proteins with corresponding molecular weights from the UniProt database. All spectra were visualized using FlexAnalysis (v3.4, Bruker Daltonics, Bremen, Germany) after smoothing and baseline subtraction.

## 3. Results

### 3.1. Genome Analysis

In total, 106 strains of *B. cereus* and 175 strains of *B. thuringiensis* (Table S2) that possessed consistent classification in NCBI, GTDB, and rMLST were selected for biomarker mining. Among the theoretical molecular weights of 53 ribosomal proteins (Table S3), 13 of these (L30, L31 type B, S20, S6, S10, L18, S13, L15, L13, S7, L6, L5, and L3) showed mass variations between *B. cereus* and *B. thuringiensis*, with a sensitivity greater than 98% (Table 1). Eleven ribosomal proteins with molecular weights below 20,000 Da could serve as potential biomarkers for MALDI-TOF MS measurements.

### 3.2. Identification of B. cereus and B. thuringiensis Based on the pycA Gene

**Figure 1:**
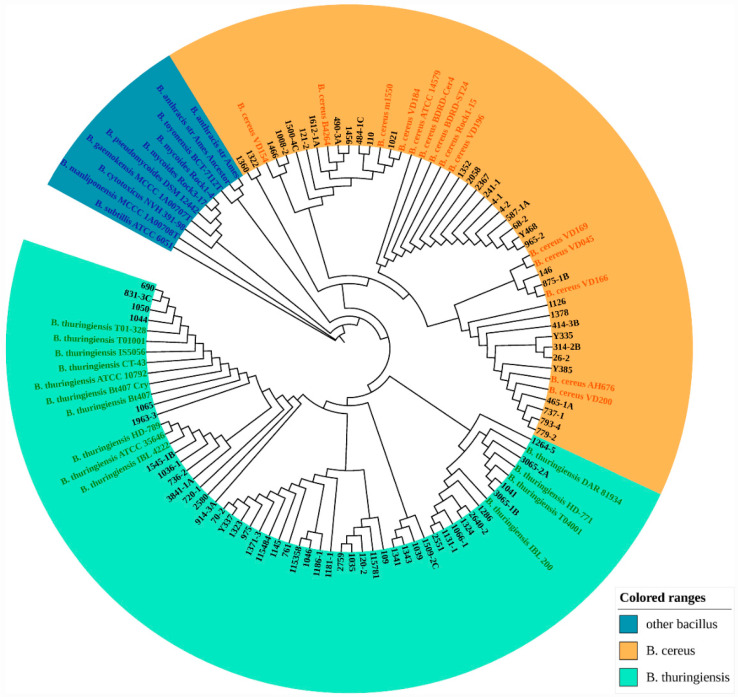
*pycA* gene-based phylogenetic tree of *Bacillus cereus*, *Bacillus thuringiensis*, and other *Bacillus cereus* group species. Branch quality was evaluated using 1000 bootstrap replicates. The reference strains are indicated in color, and the isolated strains are indicated in black.

As the phylogenetic analysis of the *pycA* gene was able to rapidly differentiate between *B. cereus* and *B. thuringiensis* [23], we used this gene as a criterion to select the correct strains for further experiments. The *pycA* amplicon sequences of *B. cereus*, *B. thuringiensis*, and reference strains were listed in Table S1. A phylogenetic tree was constructed based on the *pycA* gene (Figure 1). *B. subtilis* ATCC 6051 was used to root the tree, and the strains within the *B. cereus* group in Liu’s study were used as reference strains. Different strains of *B. cereus* or *B. thuringiensis* were clearly divided into two branches. The *pycA* gene sequence-based phylogenetic analysis assigned the isolated strains to definite *B. cereus* and *B. thuringiensis*, which were used for further MALDI-TOF MS measurements to discover potential biomarkers.

### 3.3. Discovery and Identification of Biomarkers in MALDI-TOF MS

**Figure 2:**
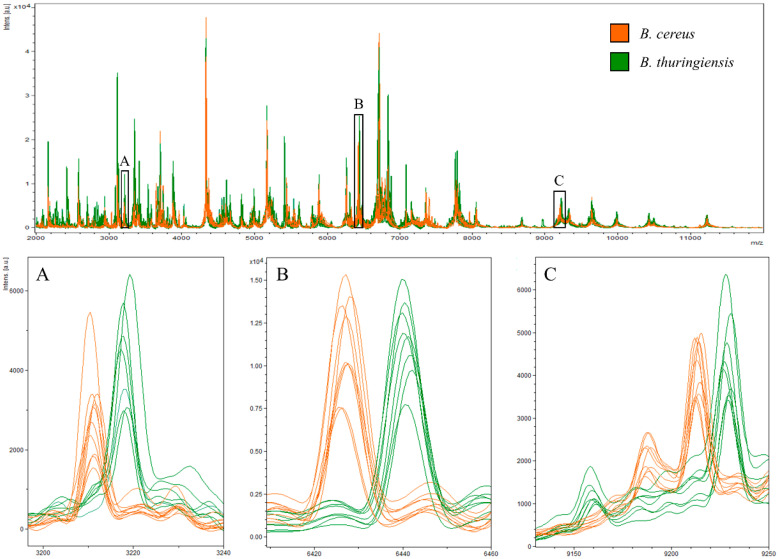
MALDI-TOF mass spectra and species-specific peaks of *Bacillus cereus* (orange) and *Bacillus thuringiensis* (green). The *y*-axis indicates peak intensities, and the *x*-axis indicates the m/z values; (**A**–**C**) represent enlarged views of species-specific peaks.

Comparative analysis of mass patterns showed that *B. cereus* and *B. thuringiensis* each possessed four species-specific peaks (Figure 2), which were highly conserved and could be considered as species-level biomarkers (Table 2). The peaks at m/z 6427 and 9214 were reproducibly found in 100% (36 of 36) *B. cereus* strains in the present study, and the peaks at m/z 3211 and 9188 were observed in 97.2% of the MS acquisitions. Meanwhile, the peaks at m/z 3218, 6441, 9160, and 9229 were detected in 100% (45 of 45) *B. thuringiensis* strains used in the present study.

By comparing the experimental m/z with genome data, the ion peaks at m/z 3211 and 3218 m/z were putatively identified as double-charged ions of the 50S ribosomal protein L30 at m/z 6427 and 6441. The peaks at m/z 9160 and 9188 were assigned to 50S ribosomal protein L31 type B, and the peaks at m/z 9214 and 9229 were characterized as 30S ribosomal protein S20. While a fraction of *B. cereus* lacked the peak at m/z 9188 representing 50S ribosomal protein L31 type B in the MALDI-TOF MS measurement (Table 2), the same result was consistently observed in the genome analysis (Table 1). Furthermore, the sequences of corresponding genes revealed non-synonymous mutations resulting amino acid substitutions in corresponding proteins, producing peak shifts from *B. cereus* to *B. thuringiensis* (Table 2).

### 3.4. Assessment of Practical Application of MALDI-TOF MS

To characterize biomarker specificity, the performance of MALDI-TOF MS targeting four biomarkers was evaluated with 13 common foodborne pathogens. Most of the other foodborne pathogens we tested did not possess the biomarkers owned by *B. cereus* or *B. thuringiensis* (Table 3 and Figure S1). The four biomarkers avoided overlapping with other common foodborne pathogenic strains and therefore achieved high specificity in routine application.

## 4. Discussion

**Figure 3:**
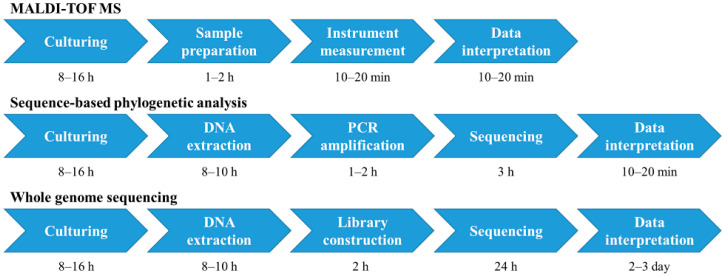
Procedures and time requirements in different methods. The time cost was calculated based on the preparation of 96 samples.

Genome-based taxonomy has become increasingly important in bacterial taxonomy and is now recognized as a more standardized taxonomic framework based on robust phylogenetic analysis than traditional classification methods [21]. However, genome-based taxonomy is time-consuming, expensive, and labor-intensive; thus, it cannot be used for fast and high-throughput microbial identification. As MALDI-TOF MS is a fast, cost-efficient, and robust approach for microbial identification, in the present study, we screened the correct *B. cereus* and *B. thuringiensis* using the GTDB and rMLST databases and transformed the robust genome variations (especially variations in ribosomal protein genes) into visible peak differences in MALDI-TOF MS, providing an easy and rapid tool for differentiating between *B. cereus* and *B. thuringiensis*. This method greatly shortened the detection time (Figure 3) and enabled high accuracy.

Several studies have been conducted on the applicability of MALDI-TOF MS for discriminating strains within *B. cereus* group. MALDI-TOF MS combined with statistical method and classifying model showing great clustering for *B. anthracis*, *B. cereus*, *B. mycoides*, *B. wiedmannii*, *B. thuringiensis*, *B. toyonensis*, and *B. weihenstephanensis* [33]. Small molecules secreted by *B. cereus* group also showed the discriminatory power, Ha pointed out that m/z at 714.2 and 906.5 can be potential biomarkers to differentiate *B. cereus* and *B. thuringiensis* [32]. Detection of specific mass peaks in the low-mass range may requires better performing instrument, which is not available in every laboratory. In the present study, we utilized the wealth of the genome data to identify ribosomal proteins that are useful for taxonomic identification, resulting in clear differentiation of *B. cereus* and *B. thuringiensis*. The species-specific peaks detected in our study were also observed in other studies, such as m/z at 6427 ± 3 and 9214 ± 3 for *B. cereus*, m/z at 6441 ± 3 and 9229 ± 3 for *B. thuringiensis* [50], showing the inter-laboratory reproducibility of this method. As one biomarker was insufficient for high-resolution taxonomy, we combined three ribosomal proteins (four specific peaks) to reliably differentiate *B. cereus* and *B. thuringiensis* with high specificity.

In this study, the genomic analysis focused on ribosomal proteins, as the corresponding genes were the basis of molecular taxonomy [51]. Conserved ribosomal proteins were highly abundant in the cytoplasm and readily detected by MALDI-TOF MS, which showed its discriminatory power by detecting slight variations in the mass of ribosomal proteins due to amino acid substitutions. The robust substitutions in the amino acid sequences were derived from non-synonymous mutations in the corresponding genes [52]. The steady accumulation of non-synonymous mutations offered genetic diversity to resolve phylogenetic relationships between isolates [53]. Recent taxonomic studies have revealed that a MALDI-TOF MS-based typing tool showed high concordance with molecular typing methods such as MLST [36,54] and core-genome analysis [34]. Tamura and co-workers developed the *S10*-GERMS (*S10*-*spc*-*alpha* operon gene encoded ribosomal protein mass spectrum) method and confirmed ribosomal subunit proteins (S10, S16, S20 and L30) could discriminate psychrotolerant species of the *B. cereus* group [50]. Fiedoruk analyzed the peak masses of *B. cereus* group and observed that ribosomal proteins L31 had the highest discriminative value to differentiate emetic *B. cereus* [55]. 

The combination of genomics and MALDI-TOF MS measurement not only transferred reliable genome-based taxonomy to a faster MALDI-TOF MS platform but also supported the interpretation of different mass patterns with objective genome data. As ribosomal proteins provide phylogenetic information and are accessible to many bacteria [56], this approach provides a framework to identify other closely related species. In the future, MALDI-TOF MS has the potential to differentiate other closely related species and phylogenetically-related strains with different levels of virulence [57], antibiotic resistance [58,59], and host origin [34].

## 5. Conclusions

By combining genomics and MALDI-TOF MS measurement, we identified four specific mass peaks for differentiating *B. cereus* and *B. thuringiensis* with high specificity. Genome-guided MALDI-TOF MS is suitable for high-throughput, cost-efficient, and reliable differentiation of *B. cereus* and *B. thuringiensis*; could serve as a first-line identification tool, and can be extended to other closely related species.

## Figures and Tables

**Table 1:** Theoretical molecular weight and sensitivity of ribosomal proteins with variations between *Bacillus cereus* and *Bacillus thuringiensis* from the genome analysis.

Gene	Annotation	Post-Translational Modification	Theoretical Molecular Weight (Da)		Sensitivity (%)	
			*B. cereus*	*B. thuringiensis*	*B. cereus*	*B. thuringiensis*
*rpmD*	50S ribosomal protein L30	methionine removed	6424.60	6438.62	100 (106/106)	100 (175/175)
*rpmE2*	50S ribosomal protein L31 type B	-	9184.33	9157.30	98.11 (104/106)	100 (175/175)
*rpsT*	30S ribosomal protein S20	methionine removed	9210.58	9226.58	100 (106/106)	99.43 (174/175)
*rpsF*	30S ribosomal protein S6	-	11,298.99	11,284.92	100 (106/106)	98.86 (173/175)
*rpsJ*	30S ribosomal protein S10	methionine removed	11,552.45	11,566.48	100 (106/106)	100 (175/175)
*rplR*	50S ribosomal protein L18	-	13,105.97	13,093.92	100 (106/106)	100 (175/175)
*rpsM*	30S ribosomal protein S13	methionine removed	13,687.83	13,661.79	100 (106/106)	100 (175/175)
*rplO*	50S ribosomal protein L15	-	15,477.74	15,521.79	100 (106/106)	100 (175/175)
*rplM*	50S ribosomal protein L13	-	16,427.96	16,457.98	100 (106/106)	100 (175/175)
*rpsG*	30S ribosomal protein S7	methionine removed	17,779.61	17,914.77	99.06 (105/106)	100 (175/175)
*rplF*	50S ribosomal protein L6	methionine removed	19,384.34	19,341.32	99.06 (105/106)	100 (175/175)
*rplE*	50S ribosomal protein L5	-	20,165.48	20,136.44	100 (106/106)	100 (175/175)
*rplC*	50S ribosomal protein L3	methionine removed	22,560.99	22,544.99	99.06 (105/106)	99.43 (174/175)

**Table 2:** Frequencies and assignments of species-specific peaks for *Bacillus cereus* and *Bacillus thuringiensis*.

Experimental m/z	Presence of Peak (%)		Protein Name	Post-Translational Modification	Theoretical m/z	Amino Acid Substitution
	*B. cereus*	*B. thuringiensis*				
3211	97.22 (35/36)	0.00 (0/45)	50S ribosomal protein L30 *	methionine removed	3213	V → L
3218	0.00 (0/36)	100.00 (45/45)			3220	
6427	100.00 (36/36)	0.00 (0/45)	50S ribosomal protein L30		6426	
6441	0.00 (0/36)	100.00 (45/45)			6440	
9160	0.00 (0/36)	100.00 (45/45)	50S ribosomal protein L31 type B	-	9158	N → S, L → I
9188	97.22 (35/36)	0.00 (0/45)			9185	
9214	100.00 (36/36)	0.00 (0/45)	30S ribosomal protein S20	methionine removed	9212	A → S
9229	0.00 (0/36)	100.00 (45/45)			9228	

* doubly charged ions.

**Table 3:** *Bacillus* strains and other common foodborne pathogens tested in the present study and MALDI-TOF MS results of specificity tests.

Bacterial Species	Strain	m/z of Biomarkers							
		3211	6427	9188	9214	3218	6441	9160	9229
*Bacillus cereus*	ATCC 14579	+	+	+	+	−	−	−	−
*Bacillus thuringiensis*	ATCC 10792	−	−	−	−	+	+	+	+
*Bacillus megaterium*	ATCC 14581	−	−	−	−	−	−	−	−
*Escherichia coli*	ATCC 25922	−	−	−	−	−	−	−	+
*Escherichia coli*	ATCC 8739	−	−	−	−	−	−	−	+
*Salmonella Enteritidis*	CMCC 50335	−	−	−	−	−	−	−	−
*Salmonella Typhimurium*	ATCC 14028	−	−	−	−	−	−	−	−
*Vibrio parahaemolyticus*	ATCC 33847	+	+	−	−	−	−	−	−
*Listeria monocytogenes*	ATCC 19115	−	−	−	−	−	−	−	−
*Staphylococcus aureus*	ATCC 25923	+	+	−	−	−	−	−	−
*Cronobacter sakazakii*	ATCC 29544	−	−	−	−	−	−	−	−
*Pseudomonas aeruginosa*	ATCC 15442	−	−	−	−	−	−	−	−
*Campylobacter jejuni*	ATCC 33291	−	−	−	−	+	−	+	−
*Yersinia enterocolitica*	CMCC 52204	−	−	−	−	−	−	−	−
*Klebsiella pneumoniae*	ATCC 700603	−	−	−	−	−	−	−	−

+/− indicate the positive and negative results, respectively.

## Data Availability

Data supporting reported results can be found in Supplementary Materials.

## Supplementary Materials

The following supporting information can be downloaded at https://www.mdpi.com/article/10.3390/microorganisms10050918/s1. Table S1. *B. cereus* and *B. thuringiensis* isolates and reference strains used in this study and identification result based on *pycA* gene sequences, Table S2. Information for *B. cereus* and *B. thuringiensis* used in genome analysis, Table S3. Theoretical molecular weight of 53 ribosomal proteins in *B. cereus* and *B. thuringiensis* from the genome analysis, Figure S1. MALDI-TOF mass spectra of *B. cereus* (orange), *B. thuringiensis* (green), and common foodborne pathogens. Orange and green arrows represent species-specific peaks of *B. cereus* and *B. thuringiensis*, respectively.
