# Supplementary figures and images for: Differentiation of Bacillus cereus and Bacillus thuringiensis Using Genome-Guided MALDI-TOF MS Based on Variations in Ribosomal Proteins
Source: Microorganisms. 2022 Apr 27;10(5):918. doi: 10.3390/microorganisms10050918 (PMC9146703; doi:10.3390/microorganisms10050918)

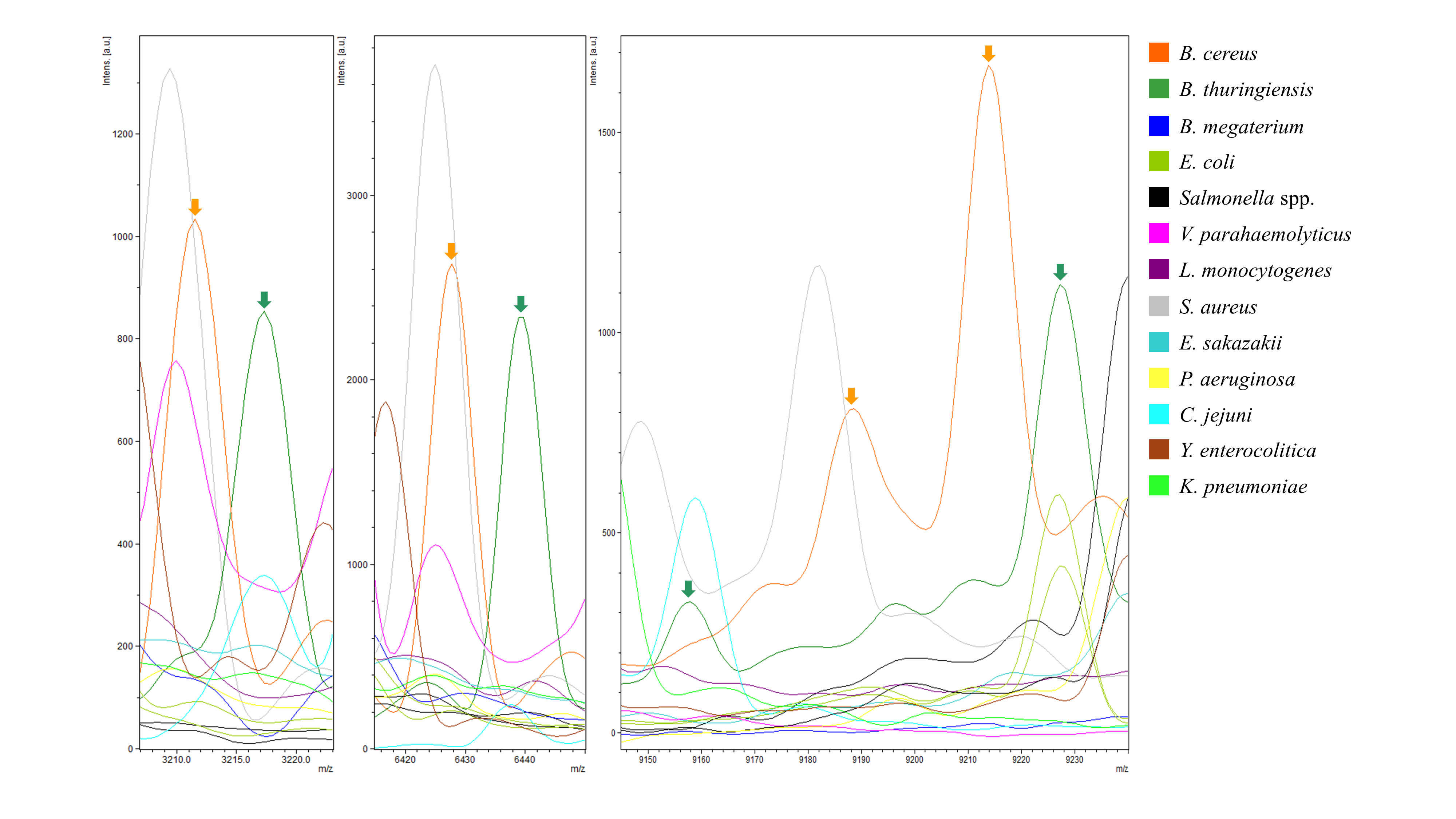

Supplement: Supplementary file 1 [file microorganisms-10-00918-s001.zip › FigureS1.tif]
